# An Evidence-Informed Clinical Pathway for COPD Exacerbations in Emergency Departments: Diagnostic Assessment, Severity Stratification, and Individualized Management

**DOI:** 10.3390/jcm15156119

**Published:** 2026-08-06

**Authors:** Rafael Suarez del Villar Carrero, Santiago Toranzo-Nieto, Laura Álvarez Santin

**Affiliations:** 1 Emergency Department, Hospital Universitario HM Montepríncipe, HM Hospitales, 28660 Madrid, Spain; 2 Centro Universitario HM Hospitales de Ciencias de la Salud, Universidad Camilo José Cela, 28692 Madrid, Spain; lalvarezsantin@hmhospitales.com; 3 Instituto de Investigación Sanitaria HM Hospitales, 28015 Madrid, Spain; 4 Emergency Department, Complejo Asistencial Universitario de Segovia, 40002 Segovia, Spain; santitoranzonieto@gmail.com; 5 Department of Pulmonology, Hospital Universitario HM Montepríncipe, HM Hospitales, 28660 Madrid, Spain

**Keywords:** chronic obstructive pulmonary disease, exacerbation, emergency department, clinical pathway, diagnostic uncertainty, severity, non-invasive ventilation, antimicrobial stewardship

## Abstract

**Background/Objectives:** Exacerbations of chronic obstructive pulmonary disease (COPD) are a common reason for emergency department (ED) attendance and hospital admission. Acute respiratory worsening is non-specific, and previous spirometric confirmation may be unavailable at presentation. We aimed to translate current recommendations into an ED-specific clinical pathway that standardizes safety-critical steps without replacing individualized clinical judgment. **Methods:** We performed a targeted synthesis of international and Spanish guidance and key studies relevant to ED diagnosis, severity assessment, pharmacological treatment, respiratory support, antimicrobial stewardship, and disposition. The evidence base was updated in June 2026, and the pathway was reviewed by a multidisciplinary clinical group. **Results:** The revised pathway comprises four stages: (1) verify previous COPD confirmation, identify an acute exacerbation-like syndrome, and assess alternative or concomitant diagnoses; (2) grade acute severity using point-of-care variables and separately assess patient vulnerability; (3) provide severity-adjusted treatment with early reassessment; and (4) determine disposition and follow-up. It incorporates controlled oxygen, individualized bronchodilator delivery, explicit criteria for systemic corticosteroids and antibiotics, assessment of risk for Pseudomonas aeruginosa, non-invasive ventilation (NIV), and a defined adjunctive role for high-flow nasal cannula (HFNC). Patients without previous post-bronchodilator spirometry are classified as having suspected COPD and require confirmation after recovery. **Conclusions:** This evidence-informed pathway is a preliminary clinical framework, not a diagnostic rule or validated decision instrument. It is intended to support, rather than replace, professional judgment. Feasibility, acceptability, diagnostic safety, and clinical impact require prospective evaluation, beginning with a pilot implementation study.

## 1. Introduction

The global burden of chronic obstructive pulmonary disease (COPD) continues to increase because of population ageing and persistent exposure to tobacco smoke, air pollution, and other risk factors [1,2,3]. COPD affects more than 200 million people worldwide and remains a major cause of morbidity, mortality, and healthcare use [1,2,3].

Exacerbations are acute episodes of respiratory worsening associated with impaired quality of life, accelerated functional decline, recurrent healthcare use, and increased short- and long-term mortality [4,5,6]. Their clinical expression is heterogeneous, and the concept of COPD exacerbation syndrome emphasizes that several inflammatory, infectious, cardiovascular, and environmental mechanisms may produce a similar acute presentation [7,8].

The burden is especially visible in EDs, where COPD exacerbations are frequent and management varies substantially between clinicians and institutions [9,10,11,12,13,14,15]. Diagnostic uncertainty is amplified by COPD underdiagnosis, incomplete access to previous records, and the frequent absence of prior post-bronchodilator spirometry [11,13,16].

GOLD 2026 defines an exacerbation as an acute event with symptoms worsening over a few days (up to 14 days), characterized by increased dyspnea and/or cough and sputum, with or without tachypnea or tachycardia [1,17]. These features are not specific. Pneumonia, acute heart failure, pulmonary embolism, pneumothorax, acute coronary syndrome or arrhythmia, and asthma may mimic, precipitate, or coexist with an exacerbation and must be assessed in parallel [1,7,8,13,16].

International and national guidelines provide comprehensive recommendations, but their application in time-constrained ED workflows may still be inconsistent [1,7,8,13,14,18,19]. A useful pathway should therefore organize minimum diagnostic and therapeutic safety checks while preserving individual decisions based on acute severity, previous treatment, comorbidity, frailty, microbiological history, patient preferences, and social circumstances.

The aim of this work was to develop an evidence-informed clinical pathway for patients presenting to the ED with confirmed or suspected COPD and acute respiratory worsening. The pathway is intended to support structured assessment, treatment, reassessment, and disposition; it is not intended to establish COPD without spirometry or to replace professional judgment.

## 2. Materials and Methods

### 2.1. Rationale for Pathway Development and Objective

The pathway was developed to translate established recommendations for COPD exacerbation management into an operational sequence adapted to ED practice [1,7,8,13,18]. Its purpose is to standardize safety-critical processes—recognition of immediate threats, diagnostic verification, differential diagnosis, severity assessment, treatment, reassessment, and disposition—rather than to standardize treatment across clinically different patients.

When previous spirometric confirmation is unavailable, early stabilization may need to proceed while COPD remains a provisional diagnosis. The revised pathway therefore distinguishes confirmed COPD from suspected COPD, requires active assessment for alternative or concomitant diagnoses, and recommends post-recovery spirometry when the diagnosis has not been established [1,7,8].

The project reorganizes recommended interventions into a structured pathway but does not generate new efficacy evidence. Accordingly, the pathway is described as evidence-informed and preliminary, and no claim of improved clinical outcomes, resource use, or admission rates is made.

### 2.2. Identification of Evidence Sources

The evidence set used for the initial pathway was collected between January and May 2025 and was updated in June 2026 during revision. Priority was given to GOLD 2026 [1], the GesEPOC recommendations on COPD exacerbation syndrome and stable COPD [7,8,18], and the Rome proposal for point-of-care exacerbation severity [17]. The operational structure of the Spanish asthma guideline (GEMA 5.5) was reviewed as a conceptual model for stepwise acute-care pathways [20].

Additional sources included ED epidemiological and quality-of-care studies [11,12,13,14,15,21], evidence on early treatment and diagnostic uncertainty [9,10,16,19,22], bronchodilator delivery devices [23], controlled oxygen [24], antibiotic stewardship [25,26,27], eosinophil-guided corticosteroid strategies [28,29,30], NIV [31], HFNC [32,33,34], and risk factors for *P. aeruginosa* or resistant organisms [35]. Reference lists of key documents were screened manually. Because the objective was operational pathway development rather than a systematic review, no PRISMA-based process or formal evidence grading was undertaken.

### 2.3. Extraction and Synthesis of Key Clinical Elements

Two emergency physicians independently reviewed the selected sources and extracted elements relevant to: (i) confirmation or suspicion of COPD; (ii) recognition of an acute exacerbation-like syndrome and alternative diagnoses; (iii) point-of-care severity assessment and baseline vulnerability; (iv) bronchodilators, corticosteroids, oxygen, antibiotics, and respiratory support; and (v) reassessment, disposition, and follow-up.

Discrepancies were resolved by consensus with a third clinician. Priority was given to variables available during the initial ED assessment and to recommendations that could be implemented without delaying resuscitation. Biomarkers were included only when they could modify a defined decision, such as C-reactive protein (CRP) for severity or stewardship in selected settings and blood eosinophils for protocolized corticosteroid-sparing strategies [17,26,29,30].

### 2.4. Integration into a Stepwise Operational Model

The extracted components were reorganized into four stages: (1) verify COPD status, identify the acute syndrome, and assess alternative or concomitant diagnoses; (2) classify acute severity and separately assess patient vulnerability; (3) initiate individualized treatment and reassess response; and (4) determine disposition and follow-up.

The framework standardizes the sequence of safety checks, not the patient. At every stage, clinicians may adapt or depart from the pathway according to the clinical context, previous and current treatment, treatment response, comorbidities, frailty, microbiological history, preferences, goals of care, and available resources.

Acute severity criteria were aligned with the Rome proposal and GOLD 2026 [1,17]. Therapeutic recommendations were aligned with current guidance and supporting studies on bronchodilator delivery, controlled oxygen, systemic corticosteroids, antibiotics, NIV, and HFNC [23,24,26,29,30,31,32,33,34]. Baseline airflow limitation, previous exacerbations, long-term oxygen or home ventilatory support, comorbidity, and social support were treated as vulnerability modifiers rather than as measures of acute episode severity [1,21,36].

### 2.5. Multidisciplinary Clinical Review

A preliminary version underwent clinical review by emergency physicians, a pulmonologist, an internist, and an ED nurse with experience in respiratory support and NIV. The review focused on internal consistency, guideline concordance, diagnostic safeguards, usability, and applicability across EDs with different resource profiles.

The revision process led to removal of the unvalidated adaptation of the CODE questionnaire, separation of acute severity from baseline vulnerability, explicit inclusion of pneumonia and other competing diagnoses, clarification of corticosteroid and antibiotic indications, addition of *P. aeruginosa* risk assessment and HFNC, and removal of intravenous salbutamol, magnesium, and inhaled corticosteroids from the acute-treatment branches.

### 2.6. Applicability Assessment and Final Pathway Refinement

Applicability was considered for EDs with variable access to blood gas analysis, rapid imaging, respiratory intermediate care, NIV, and continuous monitoring [11,14]. When a required diagnostic or support capability is unavailable, the pathway emphasizes stabilization and timely transfer rather than a lower standard of care.

No prospective usability testing, diagnostic validation, implementation study, or comparative effectiveness study was performed. The pathway therefore requires a staged evaluation beginning with a pilot study of feasibility, acceptability, completion rates, reasons for deviation, diagnostic safety, and potential unintended consequences before multicenter validation or a pragmatic comparative trial.

## 3. Results

### 3.1. Pathway Description

#### 3.1.1. Step-by-Step Structure of the Evidence-Informed Pathway

**Figure 1:**
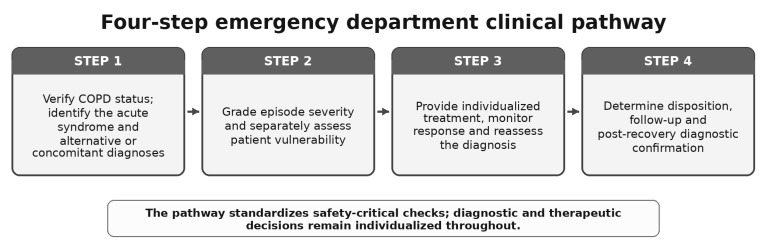
Four-step structure of the evidence-informed clinical pathway. The framework standardizes safety-critical checks while preserving individualized diagnostic and therapeutic decisions.

The revised pathway integrates four sequential but iterative decision points: diagnostic verification and differential diagnosis, acute severity and vulnerability assessment, treatment with early reassessment, and disposition with follow-up (Figure 1). Reassessment can return the clinician to an earlier step whenever the clinical course is discordant with the working diagnosis.

The pathway is designed as a clinical support framework. It does not establish COPD without post-bronchodilator spirometry, does not make treatment automatic, and does not preclude deviation when an alternative approach is clinically justified.

Step 1: Verify COPD status, identify the acute syndrome, and assess alternative diagnoses.

The first step separates three questions that are conflated in the original version: whether COPD has previously been confirmed, whether the current presentation is compatible with an acute exacerbation, and whether another condition is present. Previous post-bronchodilator spirometry or reliable specialist documentation supports confirmed COPD. In its absence, the ED diagnosis remains suspected COPD, and spirometry should be arranged after recovery. An acute exacerbation-like syndrome requires worsening over a few days (up to 14 days) of dyspnea and/or cough and sputum, with or without tachypnea or tachycardia [1,17]. Pneumonia and other alternative or concomitant diagnoses are assessed concurrently rather than sequentially (Table 1).

Step 2: Classify acute severity and assess vulnerability.

Acute episode severity is classified using the Rome point-of-care variables: dyspnea intensity on a 0–10 visual analogue scale (VAS), respiratory rate, heart rate, resting oxygen saturation relative to room air or the patient’s usual oxygen prescription, CRP when available, and arterial blood gases when clinically indicated [1,17]. Baseline disease severity and vulnerability influence monitoring and disposition but do not define the severity of the acute episode (Table 2).

If oxygen is required, it should be started at the lowest effective concentration and titrated to a target SpO_2_ of 88–92%, with repeated clinical assessment and blood gas analysis when hypercapnia, acidosis, deterioration, or ventilatory support is suspected [1,24]. Oxygen should also be titrated during NIV. The alveolar–arterial oxygen gradient is not included as a routine decision criterion because current acute-care pathways base escalation primarily on oxygenation, PaCO_2_, pH, work of breathing, mental status, and response to treatment [1,31].

Step 3: Provide individualized treatment and reassess.

Previous and prehospital inhaled treatment, cumulative short-acting bronchodilator doses, maintenance therapy, inhaler technique, heart rate, arrhythmias, and clinical response should be reviewed before additional dosing. A short-acting beta_2_-agonist (SABA), with or without a short-acting muscarinic antagonist (SAMA), is recommended initially for moderate or severe exacerbations [1]. A pressurized metered-dose inhaler (pMDI) with spacer is preferred when the patient can use it effectively. Nebulization remains appropriate when coordination is poor, dyspnea is severe, or ventilatory support is required; if used, an air-driven nebulizer is preferred and oxygen should be titrated separately [1,23]. Repeated dosing is guided by response and adverse effects rather than by a fixed high-dose schedule.

Systemic corticosteroids are recommended for moderate or severe exacerbations with clinically significant worsening. A prednisone-equivalent dose of 40 mg once daily for 5 days is suggested, with the oral route preferred when feasible [1]. They are not presented as automatic treatment for every mild presentation. Blood eosinophils may support corticosteroid-sparing protocols in selected, non-life-threatening patients, but current trial thresholds differ and the count should not be used as the sole reason to withhold corticosteroids in severe or life-threatening presentations [28,29,30].

Antibiotics are recommended when at least two of the following are present—greater dyspnea, fever, increased sputum volume, and increased sputum purulence—provided purulence is one of the findings; when a relevant previous sputum culture was positive; or when invasive or non-invasive mechanical ventilation is required [1]. Pneumonia and other bacterial infections should be managed according to their specific guidelines. Choice, route, and duration should reflect local resistance patterns, previous cultures, recent antibiotic exposure, allergy, renal function, severity, and oral treatment capacity (Table 3) [1,25,26,27].

NIV is the preferred initial ventilatory support for acute hypercapnic respiratory failure with acidosis or persistent increased work of breathing despite initial treatment [1,31]. HFNC may be considered for persistent hypoxemia despite conventional oxygen, during selected NIV breaks, or when NIV is not tolerated or is contraindicated, provided monitoring and explicit failure criteria are available. Evidence is evolving, and HFNC must not delay NIV or intubation when indicated [1,32,33,34]. Intravenous salbutamol and magnesium are not included in routine COPD exacerbation treatment.

Before discharge or shortly after stabilization, maintenance inhaled therapy should be reviewed, including adherence and technique. Long-acting bronchodilator therapy should be continued or initiated as indicated. Inhaled corticosteroids are considered only as part of maintenance treatment—usually with long-acting bronchodilators—when supported by asthma, exacerbation history, and blood eosinophils; they are not an acute substitute for systemic corticosteroids [1,18].

Step 4: Determine disposition and follow-up.

Disposition is based on response to initial treatment, gas exchange, respiratory support requirements, unresolved diagnoses, comorbidity, frailty, baseline support needs, treatment capacity at home, and patient preferences (Table 4). Life-threatening features, NIV failure, invasive ventilation, shock, refractory hypoxemia, severe or worsening acidosis, or significant mental-status change require urgent respiratory intermediate-care or intensive-care assessment [1,21,31].

Patients discharged from the ED should have clear return precautions and early clinical reassessment, generally within 48–72 h according to local pathways and clinical risk. The follow-up plan should address symptom trajectory, oxygen requirement, medication tolerance, inhaler technique, adherence, smoking cessation, and the need to revise the working diagnosis [19].

Patients without previous diagnostic-quality post-bronchodilator spirometry should undergo spirometric confirmation after the acute episode has resolved. Specialist review should be prioritized when diagnostic uncertainty persists, exacerbations are recurrent, respiratory support is required, or major comorbidity complicates management.

#### 3.1.2. Intended Use and Clinical Safeguards

The pathway standardizes a sequence of minimum safety checks; it does not prescribe identical care. Individual treatment is modified by prior and current therapy, phenotype, comorbidity, frailty, microbiology, adverse-effect risk, response to initial interventions, goals of care, and social context.

**Figure 2:**
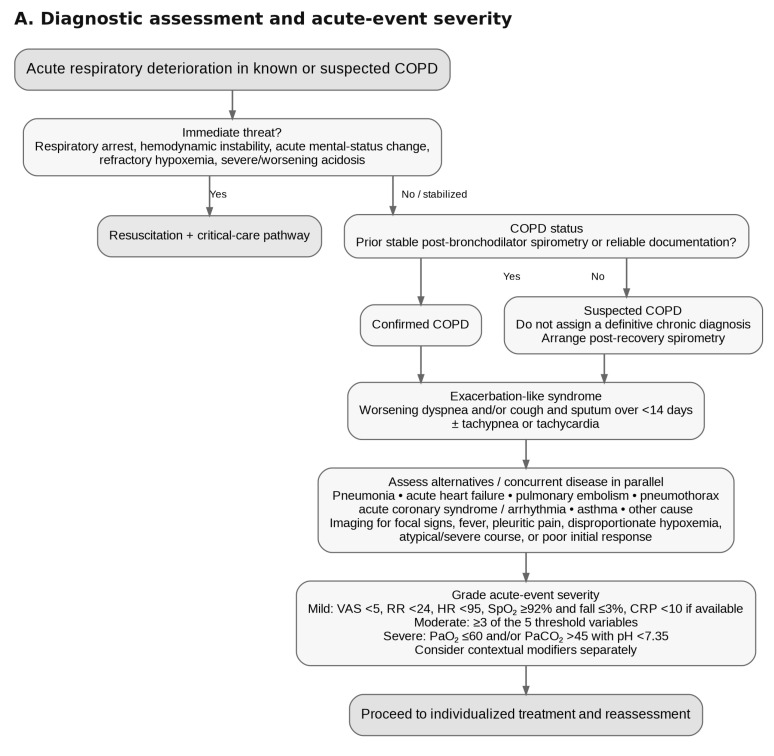

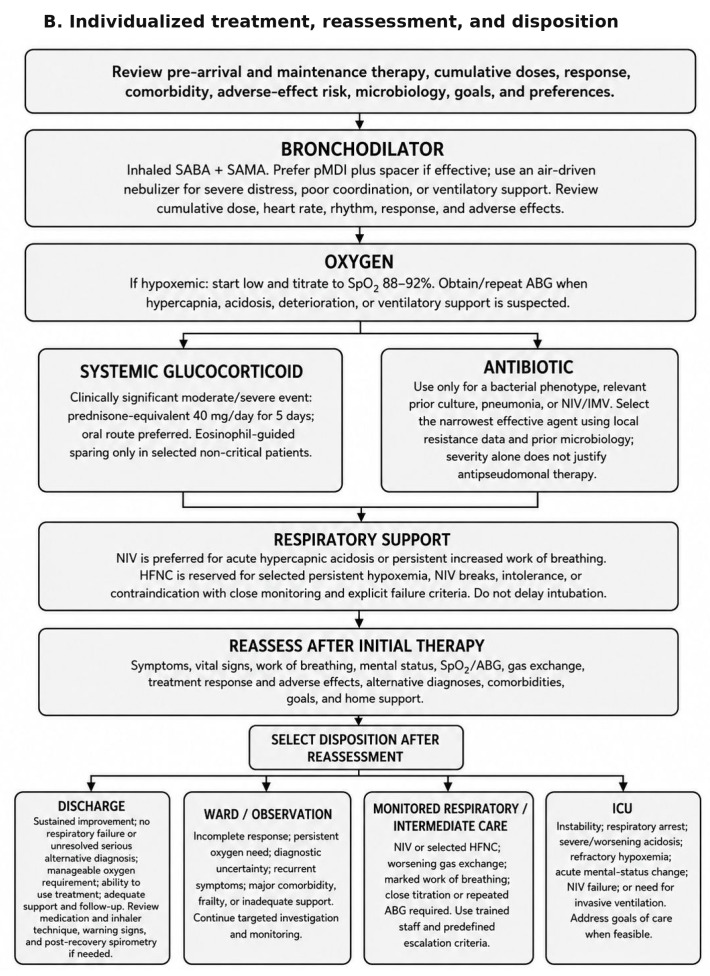
Evidence-informed clinical pathway for an acute COPD exacerbation-like presentation in the ED: (**A**) diagnostic assessment and acute-event severity; (**B**) individualized treatment, reassessment, and disposition. ABG, arterial blood gas; HFNC, high-flow nasal cannula; IMV, invasive mechanical ventilation; NIV, non-invasive ventilation.

Figure 2 summarizes the revised diagnostic and therapeutic pathway. Diagnostic uncertainty is maintained explicitly when COPD has not been confirmed, pneumonia is incorporated as a potentially concomitant diagnosis, and repeated reassessment is required before disposition.

The pathway has not been tested prospectively. Its proposed benefits—such as greater consistency or faster recognition of deterioration—remain hypotheses and should not be interpreted as demonstrated improvements in outcomes, workload, resource use, or hospital admission rates [14,15].

## 4. Discussion

Although age-standardized COPD mortality has declined in several regions, the absolute burden continues to rise, and exacerbations remain strongly associated with functional decline, recurrent events, cardiovascular complications, healthcare use, and mortality [1,2,3,4,5,13,25,37,38,39]. ED care must reconcile rapid stabilization with diagnostic uncertainty and substantial variation in resources and practice [12,13,14,15].

The main contribution of this work is not a new diagnostic test or treatment. It is the operational reorganization of existing recommendations into an ED pathway that explicitly separates COPD confirmation, recognition of the acute syndrome, differential diagnosis, acute severity, baseline vulnerability, treatment, reassessment, and disposition. This distinction addresses a major weakness of the original version, in which a screening questionnaire could be interpreted as a shortcut to diagnosis.

### 4.1. Standardizing Safety-Critical Processes Without Uniform Treatment

Standardization is most defensible when applied to processes that should not be omitted: identifying immediate threats, checking whether COPD has been confirmed, considering pneumonia and other serious alternatives, measuring severity, reviewing previous treatment, titrating oxygen, reassessing response, and documenting disposition criteria. It is not defensible when it forces the same treatment on patients with different phenotypes, comorbidities, adverse-effect risks, microbiological histories, or preferences.

The revised pathway therefore treats acute severity and baseline vulnerability as related but distinct dimensions. A physiologically mild acute event may still warrant admission because of frailty, severe baseline disease, repeated recent exacerbations, long-term oxygen or home NIV, major comorbidity, or inadequate support. Conversely, a patient should not receive broader antibiotics, repeated high-dose SABA, or an inhaled corticosteroid during the acute phase merely because the pathway has reached a particular box [1,21,36].

Implementation will require local adaptation, education, access to respiratory support, and audit of deviations and adverse events. A pathway can reduce omissions only if clinicians understand its scope and retain authority to override it. Algorithmic neatness is not a clinical outcome; concordance with the patient’s actual problem is.

### 4.2. Diagnostic and Therapeutic Safeguards

The diagnostic safeguard is deliberate retention of uncertainty. Spirometry remains necessary to establish COPD, but it is generally not a test for the unstable acute phase [1]. Patients without previous confirmation are therefore labelled as suspected COPD, treated according to immediate physiological needs, and scheduled for post-recovery confirmation. The differential diagnosis includes pneumonia, heart failure, pulmonary embolism, pneumothorax, acute coronary syndrome or arrhythmia, and asthma, with imaging and additional tests guided by clinical findings [1,7,8].

Therapeutic safeguards include review of cumulative bronchodilator exposure; preference for pMDI with spacer when feasible without prohibiting nebulization in patients unable to use handheld devices; controlled oxygen to SpO_2_ 88–92%; defined indications for systemic corticosteroids and antibiotics; and selection of antibiotic spectrum according to local epidemiology and individual risk rather than acute severity alone [1,23,24,26,27,35].

Blood eosinophils can support a more personalized corticosteroid strategy, but CORTICO-COP and STARR2 studied different settings and thresholds, so universal withholding rules are not justified [29,30]. NIV remains first-line ventilatory support for acute hypercapnic respiratory failure with acidosis. HFNC is now included because randomized evidence has expanded, but treatment failure and crossover to NIV remain clinically important; close monitoring and prompt escalation are essential [1,31,32,33,34].

These revisions improve face validity and guideline concordance, but they do not establish effectiveness. Whether the pathway changes diagnostic accuracy, treatment exposure, time to appropriate support, admission, relapse, readmission, or mortality remains unknown.

### 4.3. Study Limitations and Research Agenda

This work has several limitations. The evidence search was targeted rather than systematic, evidence certainty was not formally graded, and the multidisciplinary review was not a formal Delphi process. The pathway may also embed assumptions from Spanish and European practices that are not transferable to all healthcare systems.

The pathway has not been prospectively tested for usability, inter-rater consistency, diagnostic misclassification, clinician adherence, or unintended consequences. Some decision points depend on blood gas analysis, imaging, continuous monitoring, NIV, or HFNC, and institutions without these capabilities will require explicit transfer pathways. The Rome thresholds and biomarker-guided strategies also require further validation across ED populations and disease severities [17,29,30].

The next step should be a pilot implementation study evaluating acceptability to ED clinicians, completion rates, time burden, reasons for deviation, diagnostic safety, treatment exposure, and prespecified escalation failures. The pathway should then be refined and evaluated prospectively across multiple centers. Only after feasibility and safety are demonstrated should a pragmatic or cluster-randomized comparison assess patient-important outcomes and resource use.

## 5. Conclusions

The proposed pathway is a structured, evidence-informed framework for the evaluation and individualized management of patients with confirmed or suspected COPD who present to the ED with acute respiratory worsening. It is not a diagnostic rule or a clinically validated decision instrument and does not replace professional judgment. Its feasibility, acceptability, diagnostic safety, and effect on care processes and clinical outcomes require staged prospective evaluation, beginning with a pilot implementation study.

## Figures and Tables

**Table 1:** Operational diagnostic framework for an acute COPD exacerbation-like presentation.

Domain	Operational Assessment	Action
COPD status	Is there previous post-bronchodilator spirometry showing persistent airflow obstruction or reliable specialist documentation?	Yes: confirmed COPD. No: suspected COPD; avoid a definitive ED diagnosis and arrange spirometry when clinically stable.
Acute syndrome	Dyspnea and/or cough and sputum worsening over a few days (up to 14 days), with or without tachypnea or tachycardia?	If present, assess severity and precipitating factors. If absent or atypical, broaden the differential diagnosis.
Alternative or concomitant diagnosis	Focal chest findings, fever, pleuritic pain, disproportionate hypoxemia, edema, chest pain, arrhythmia, hemoptysis, unilateral breath sounds, or poor treatment response?	Evaluate for pneumonia, heart failure, pulmonary embolism, pneumothorax, acute coronary syndrome/arrhythmia, and asthma; use imaging, ECG, and biomarkers as indicated.
Immediate threats	Respiratory arrest, shock, severe altered mental status, inability to protect the airway, refractory hypoxemia, or severe/worsening acidosis?	Initiate resuscitation and urgent critical-care and ventilatory assessment.

COPD, chronic obstructive pulmonary disease; ED, emergency department.

**Table 2:** Acute COPD exacerbation severity and separate vulnerability modifiers.

Category or Domain	Point-of-Care Criteria
Mild acute episode	Dyspnea VAS < 5; respiratory rate < 24/min; heart rate < 95/min; resting SaO_2_ ≥ 92% on room air or the patient’s usual oxygen prescription and change ≤3% when known; CRP < 10 mg/L if available.
Moderate acute episode	At least 3 of 5: dyspnea VAS ≥ 5; respiratory rate ≥ 24/min; heart rate ≥ 95/min; resting SaO_2_ < 92% on room air or usual oxygen and/or change >3%; CRP ≥ 10 mg/L if available. If obtained, ABG may show PaO_2_ 70–80 mmHg.
Severe acute episode	Moderate clinical variables plus, if ABG is obtained, PaO_2_ ≤ 60 mmHg and/or PaCO_2_ > 45 mmHg with pH < 7.35.
Immediate critical-care flags	Respiratory arrest; shock or hemodynamic instability; severe altered mental status or inability to protect the airway; refractory hypoxemia; severe or worsening acidosis; failure of initial ventilatory support.
Vulnerability modifiers (not acute severity criteria)	Severe baseline airflow limitation; long-term oxygen or home NIV; frequent or recent severe exacerbations/hospitalization; major cardiac, renal, hepatic, or other comorbidity; frailty; impaired self-management; limited home support.

ABG, arterial blood gas; CRP, C-reactive protein; FEV_1_, forced expiratory volume in one second; NIV, non-invasive ventilation; PaCO_2_, arterial carbon dioxide pressure; PaO_2_, arterial oxygen pressure; SaO_2_, arterial oxygen saturation; VAS, visual analogue scale.

**Table 3:** Antibiotic decision-making and assessment of risk for Pseudomonas aeruginosa or resistant organisms.

Decision Point	Criteria	Recommended Action
Indication for antibiotics	At least 2 of increased dyspnea, fever, increased sputum volume, and increased sputum purulence, provided purulence is one criterion; relevant previous positive sputum culture; or need for invasive/non-invasive mechanical ventilation.	Use antibiotics only when criteria are met. Point-of-care CRP may support stewardship in selected settings but does not replace clinical assessment.
Pneumonia or another bacterial infection	Clinical or imaging evidence of pneumonia or another bacterial focus.	Follow the relevant disease-specific guideline and local pathway rather than the COPD-exacerbation table alone.
Risk of *P. aeruginosa* or resistant organisms	Previous *P. aeruginosa*/MDR isolation; bronchiectasis; severe airflow obstruction; frequent exacerbations; recent hospitalization or antibiotics; repeated systemic corticosteroids; mechanical ventilation.	Obtain a respiratory sample when feasible and choose empirical therapy according to prior microbiology and local resistance patterns. Severity alone is insufficient for antipseudomonal coverage.
Choice, route, duration, and review	Consider local antibiogram, previous cultures, allergy, renal function, clinical severity, and ability to take oral therapy.	Use the narrowest effective agent; prefer oral therapy when appropriate; generally use short courses (≤5 days in outpatients and commonly 5–7 days in selected hospitalized patients); review cultures and de-escalate.

Antibiotic selection should follow local antimicrobial guidance. Broad-spectrum or antipseudomonal treatment is not indicated solely because an exacerbation is clinically severe.

**Table 4:** Suggested disposition and follow-up criteria.

Destination	Suggested Criteria and Actions
ED discharge	Clinical improvement after initial treatment; no unresolved serious alternative diagnosis; oxygen requirement at or near a manageable baseline; no respiratory acidosis or need for ventilatory support; ability to use prescribed treatment; reliable support, follow-up, and return precautions.
Hospital ward admission	Persistent or worsening dyspnea, hypoxemia, hypercapnia, or acidosis; oxygen requirement above baseline; inadequate response; need for HFNC or close monitoring; major comorbidity or complication; frailty; inability to manage treatment or insufficient home support.
Respiratory intermediate care or ICU	Life-threatening features; NIV failure; need for invasive ventilation; shock; severe/worsening acidosis; significant altered mental status; refractory hypoxemia; need for continuous advanced respiratory monitoring.
Follow-up and diagnostic confirmation	Reassess within 48–72 h according to risk and local pathways; review inhaled maintenance treatment, technique, adherence, smoking, and action plan; arrange post-recovery spirometry when COPD has not been confirmed.

## Data Availability

The original contributions presented in this study are included in the article. Further inquiries can be directed to the corresponding author.

## Abbreviations

The following abbreviations are used in this manuscript:

ABG	Arterial blood gas
ACS	Acute coronary syndrome
COPD	Chronic obstructive pulmonary disease
CRP	C-reactive protein
ED	Emergency department
FEV_1_	Forced expiratory volume in one second
GOLD	Global Initiative for Chronic Obstructive Lung Disease
GesEPOC	Spanish COPD guidelines
HFNC	High-flow nasal cannula
ICU	Intensive care unit
ICS	Inhaled corticosteroid
LABA	Long-acting beta_2_-agonist
LAMA	Long-acting muscarinic antagonist
NIV	Non-invasive ventilation
PaCO_2_	Arterial carbon dioxide pressure
PaO_2_	Arterial oxygen pressure
pMDI	Pressurized metered-dose inhaler
SABA	Short-acting beta_2_-agonist
SAMA	Short-acting muscarinic antagonist
SpO_2_	Peripheral oxygen saturation
VAS	Visual analogue scale
